# Reality = Relevance? Insights from Spontaneous Modulations of the Brain's Default Network when Telling Apart Reality from Fiction

**DOI:** 10.1371/journal.pone.0004741

**Published:** 2009-03-11

**Authors:** Anna Abraham, D. Yves von Cramon

**Affiliations:** 1 Department of Cognitive Neurology, Max Planck Institute for Human Brain and Cognitive Sciences, Leipzig, Germany; 2 Department of Clinical Psychology, Justus Liebig University of Giessen, Giessen, Germany; 3 Max Planck Institute for Neurological Research, Cologne, Germany; University of Granada, Spain

## Abstract

**Background:**

Although human beings regularly experience fictional worlds through activities such as reading novels and watching movies, little is known about what mechanisms underlie our implicit knowledge of the distinction between reality and fiction. The first neuroimaging study to address this issue revealed that the mere exposure to contexts involving real entities compared to fictional characters led to engagement of regions in the anterior medial prefrontal and posterior cingulate cortices (amPFC, PCC). As these core regions of the brain's default network are involved during self-referential processing and autobiographical memory retrieval, it was hypothesized that real entities may be conceptually coded as being more personally relevant to us than fictional characters.

**Methodology/Principal Findings:**

In the present functional magnetic resonance imaging (fMRI) study, we directly test the hypothesis that entity-associated personal relevance is the critical factor underlying the differential engagement of these brain regions by comparing the brain's response when processing contexts involving family or friends (high relevance), famous people (medium relevance), or fictional characters (low relevance). In line with predictions, a gradient pattern of activation was observed such that higher entity-associated personal relevance was associated with stronger activation in the amPFC and the PCC.

**Conclusions/Significance:**

The results of the study have several important implications. Firstly, they provide informed grounds for characterizing the dynamics of reality-fiction distinction. Secondly, they provide further insights into the functions of the amPFC and the PCC. Thirdly, in view of the current debate related to the functional relevance and specificity of brain's default network, they reveal a novel approach by which the functions of this network can be further explored.

## Introduction

One rarely comes across a person who does not enjoy engaging in fictional worlds via mediums such as television, books, computer games and pretend play. Imparting social knowledge and achieving empathic growth are some of the reasons why we universally engage in such forms of recreation which involve simulations of alternate realities from a safe vantage point [1], [2]. It is therefore fascinating that we rarely confuse fiction with reality although we can be intensely engaged in fictional worlds. Indeed, by the age of five, children already possess an intricate understanding of the reality-fiction distinction [3], [4]. Understanding the divide between reality and the more broadly construed realm of fantasy has for long been a subject of exploration in the field of developmental psychology [3], [4]. There are a several avenues therein that are broadly relevant to the current study such as the principles that guide the formation of fictional world knowledge [5], factors that enable categorization of reality-fantasy phenomena [6], and intervening variables in the understanding of the reality-fiction distinction, such as the influence of emotion and relatedness of the fictional events to real life [4], [7]. What have yet to be fully uncovered are the factors that modulate our implicit knowledge of the distinction between what is real and unreal.

In the first attempt to tackle this issue using functional magnetic resonance imaging (fMRI), we aimed to uncover which brain regions were preferentially engaged when processing either real or fictional scenarios [8]. The findings demonstrated that processing contexts containing real people (e.g., George Bush) compared to contexts containing fictional characters (e.g., Cinderella) led to activations in the anterior medial prefrontal cortex (amPFC) and the posterior cingulate cortex (PCC).

These findings were intriguing for two reasons. First, the identified brain areas have been previously implicated in self-referential thinking and autobiographical memory retrieval [9], [10]. This suggested that information about real people, in contrast to fictional characters, may be coded in a manner that leads to the triggering of automatic self-referential and autobiographical processing. This led to the hypothesis that information about real people may be coded in more personally relevant terms than that of fictional characters. We do, after all, occupy a common social world and have a wider range of associations in relation to famous people. These may be spontaneously triggered and processed further when reading about them. A logical extension of this premise would be that explicitly self-relevant information should therefore elicit such processing to an even greater extent.

Indirect support for this idea comes from other studies which have, for instance, demonstrated the engagement of the PCC when viewing social interactions between real people relative to identical scenarios performed by animated agents [11] as well as when participants played interactive games against human partners relative to computer partners [12]. Moreover, anterior mPFC and the PCC have also been reported when recalling real events relative to imagining fictitious events [13]. Even in the field of social perception where differences in the level of agent realism (e.g., humans versus robots) have received attention [e.g.], [14,15], familiarity effects have been reported in mPFC and PCC regions as a function of viewing highly familiar faces relative to less familiar faces [16].

Second, the amPFC and PCC are also considered to constitute core regions of the “default network” of the brain [17]. This network refers to a group of brain regions that are customarily more engaged during passive periods within experiments, such as at rest or when performing cognitively undemanding tasks compared to highly demanding tasks [17], [18]. Assessments of the thought content during such passive periods have revealed a preponderance of reflections concerning past events, planning of future events and self-referential mentation [19], [20]. This observation fits well with findings showing stronger activations in these regions during tasks that actively necessitate such operations [21]–[26].

The aforementioned finding of activity in default network regions being modulated by the type of semantic representation (real>fictional) [8] hinted at a means by which passive and active approaches to study the functional significance of the default network could be integrated within one paradigm. This could be optimally executed by contrasting contexts that are comparable in terms of task demands but different in that one context contains information that is more likely to spontaneously trigger active internally-directed mentation. A good candidate for such a trigger is self-relevant information as there is evidence to indicate that our attentional system is particularly sensitive to self-relevant stimuli, and that such stimuli automatically induce mind wandering [27].

**Figure 1:**
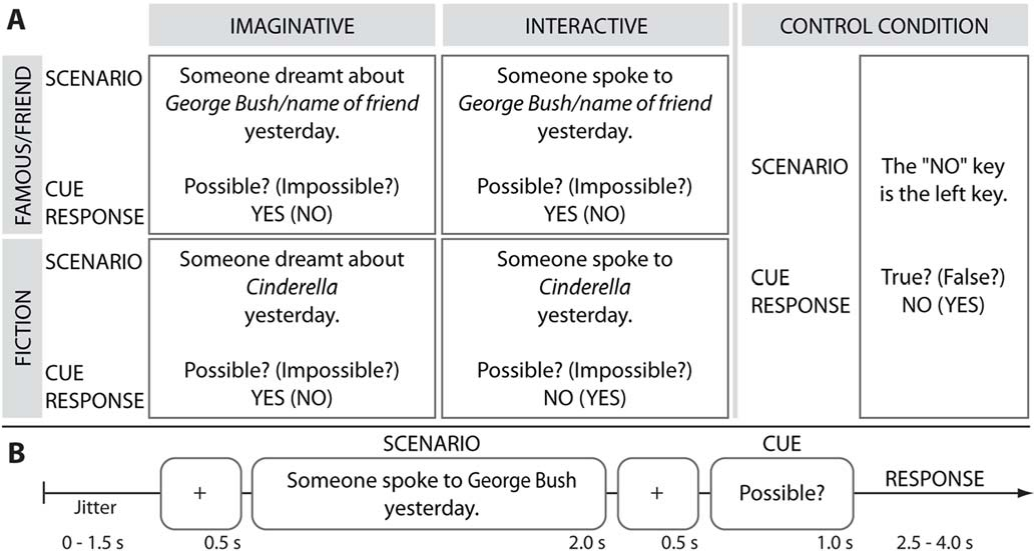
Experimental Task and Design. (a) Examples of scenarios, cues and correct responses to cues for all conditions. (b) A schematic representation of the sequence of events in a trial (trial length: 8 s). Across all experimental conditions, each trial began with a fixation cross (duration: 500 ms) which was followed by the presentation of single sentence for 2000 ms where a scenario was introduced. Following a delay (500 ms), a question cue was presented, to which the participant was required to respond. The cue remained on the computer screen for 1000 ms and the subject responded (yes or no) by pressing the appropriate button (index or middle finger) on a response box placed under the right hand. Variable jitter times were inserted before and after the scenario to enhance the temporal resolution of the blood oxygenation level-dependent (BOLD) signal. For the baseline rest period, a blank screen was presented for the duration of a trial.

By comparing the processing of high, medium and low personal relevance contexts, two expectations could be verified. First, in the context of the reality-fiction distinction, we predicted that associated personal relevance represents a critical factor that modulates automatic engagement of the amPFC and PCC. In line with this, we expected a gradient activation profile in these regions such that they would be most strongly engaged during high relevance contexts (e.g., involving one's mother), moderately engaged in medium relevance contexts (e.g., involving George Bush) and least engaged in low relevance contexts (e.g., involving Cinderella). Second, in the context of the default network, such a modulation would be evidence of a novel approach by which the responsiveness of default network could be indirectly manipulated. We investigated these questions using event-related fMRI where the experimental design involved having participants read and judge scenarios in which a real protagonist is involved in imaginative or interactive contexts together with either a fictional character (low relevance), a real person who is famous (medium relevance), or a real person who was a friend or family member of the participant (high relevance) (Figure 1).

The present findings confirm that the amPFC and the PCC are modulated by the degree of stimulus associated personal relevance. In addition, the results suggest that the current approach is promising with regard to targeting the responsiveness of the default network.

## Results and Discussion

### Behavioral results

**Figure 2:**
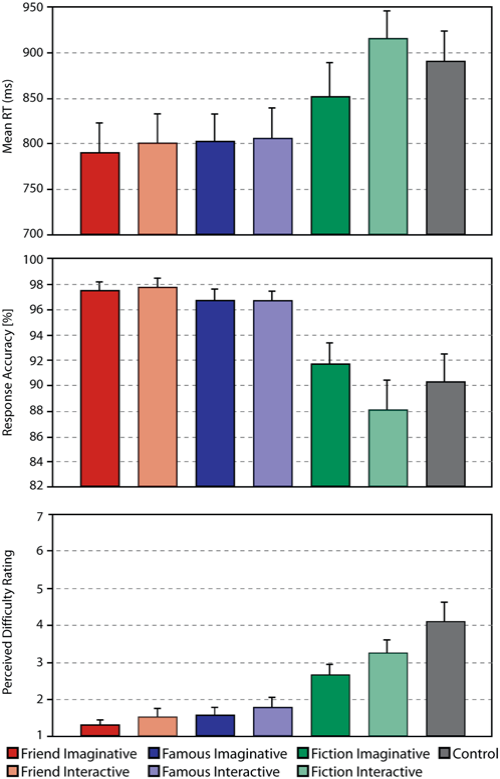
Behavioral Findings. The graphs display the results associated with each of the behavioral measures: reaction time (in milliseconds, top panel), response accuracy (in percentage of correct responses, middle panel) and perceived difficulty (in feedback ratings, bottom panel).

In order to determine the behavioral comparability between the different conditions, three behavioral indices were recorded. Two indices (Reaction time, Response Accuracy) were collected over the course of the experiment and one index (Perceived Difficulty) was assessed in the post-fMRI feedback session. Figure 2 and Table S1 of the Supporting Information (SI) show the findings associated with the behavioral measures. Only the immediately relevant behavioral findings are reported here. For further details, please refer to the Supporting Results S1 in the SI.

With regard to reaction time (RT), the type of entity to be processed was found to play a significant role (*Repeated Measures ANOVA*: *F*_2, 17_ = 109.44, *P*<.001; partial-eta squared/h_p_^2^ = 0.899), such that participants were slower when responding to scenarios involving fictional characters compared to those involving famous entities (*F*_1, 18_ = 107.75, *P*<.001, h_p_^2^ = 0.857) and friend entities (*F*_1, 18_ = 160.53, *P*<.001, h_p_^2^ = 0.899). In contrast, the RTs for scenarios involving famous and friend entities were comparable (*F*_1, 18_ = 3.82, *P*>.05). Furthermore, compared to the control condition, participants responded faster to all friend and famous conditions (*Paired samples t-test*: all *t*_18_>−4.18, all *P*<.005). The differences between the control condition and the fiction conditions were, however, non-significant (*all P*>.05).

The type of entity also had a comparable effect on response accuracy (*Repeated Measures ANOVA*: *F*_2, 17_ = 23.80, *P*<.001; h_p_^2^ = 0.569) such that response accuracy was lower when responding to scenarios involving fictional characters compared to famous (*F*_1, 18_ = 22.03, *P*<.001, h_p_^2^ = 0.55) and friend entities (*F*_1, 18_ = 29.16, *P*<.001, h_p_^2^ = 0.618). In contrast, there was no significant difference between the response accuracy of famous and friend scenarios (*F*_1, 18_ = 2.4, *P*>.05). Also, compared to the control condition, participants responded more accurately to all friend and famous conditions (*Paired samples t-test*: all *t*_18_>2.9, all *P*<.01). The differences between the control condition and the fiction conditions, however, were non-significant (all *P*>.05).

A similar pattern of findings was also found on the measure of Perceived Difficulty. Higher perceived difficulty was associated with the fiction conditions compared to the friend and famous conditions, (*Wilcoxon Signed Ranks Test*: all *Z*>2, all *P*<.05). The friend and famous conditions were, in contrast, not significantly differentiable from one another (all *P*>.05). Also, relative to the control condition, perceived difficulty was lower for all experimental conditions (all *Z*>2.6, all *P*<.01) except the fiction-interactive condition (*P*>.05).

In summary, the statistical analyses of all the behavioral measures indicate that the fiction and control conditions were comparable as lengthier RTs, lower PCR and higher perceived difficulty were associated with these conditions relative to the friend and famous conditions. The friend and famous conditions were comparable as they did not significantly differ on any of the behavioral measures.

### fMRI results

While no region of the brain was found to be significantly engaged as a function of context type (Interactive versus Imaginative; Supporting Results S1 in SI), the type of entity/character to be processed led to the differential engagement of core regions of the default mode network [17], such as the amPFC and PCC, as well as the hippocampal formation, lateral temporal cortex, and dorsal medial prefrontal cortex (Tables S2, S3 and S4 in SI).

**Figure 3:**
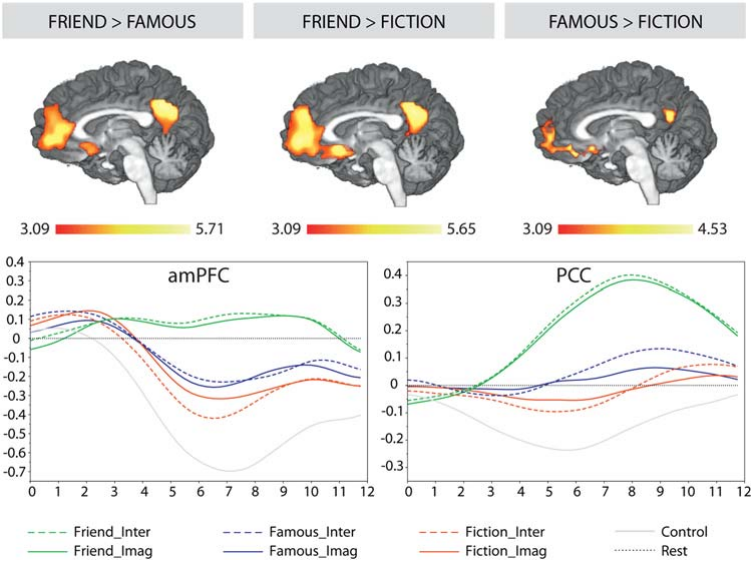
Gradient Relevance Pattern. The top panel shows activations in and around the anterior mPFC and the PCC (x = −3) as a function of the indicated contrast. The left top panel shows the Friend>Famous contrast (inclusive mask: Friend>Control), the middle top panel shows the Friend>Fiction contrast (inclusive mask: Friend>Control) and the right top panel shows the Famous>Fiction contrast (inclusive mask: Famous>Control). All reported activations were corrected for multiple comparisons (p<.05) by employing cluster-size thresholding using Monte-Carlo simulations (initial height threshold: z = 3.09). Note that activations along the frontomedian wall extend into the dorsal medial PFC only in the Friend>Famous and Friend>Fiction contrasts. The bottom panel shows the averaged percentage signal change (PSC) response associated with all conditions within a peak voxel and its 26 adjacent neighboring voxels in the anterior medial prefrontal cortex (peak voxel: −5, 49, 0) (left bottom panel) and PCC (peak voxel: −5, −56, 30) (right bottom panel). The zero point in the graphs represents the resting baseline.

In particular, in line with our predictions, regions in and near the amPFC (including the ventral mPFC) and PCC (including the retrosplenial cortex) were modulated by the degree of personal relevance associated with the presented entities (Figure 3). These regions were most strongly engaged when processing high personal relevance contexts (friend-real), secondarily for medium relevance contexts (famous-real) and least of all in the low personal relevance contexts (fiction) (high relevance>medium relevance>low relevance).

The amPFC and PCC regions are known to be commonly engaged during autobiographical and episodic memory retrieval [10], [28], [29] as well as during self-referential processing [9]. Regarding their specific roles, there is evidence indicating that amPFC is comparatively more selective for self-referential processing whereas the PCC/RSC is more selective for episodic memory retrieval [25]. The results of the present study contribute to the understanding of processes implemented in these regions by showing that the demands on autobiographical retrieval processes and self-referential mentation are affected by the degree of personal relevance associated with a processed scenario. It should additionally be noted that the extension of the activations in anterior and ventral PFC regions into subgenual cingulate areas (Figure 3) indicates that the degree of personal relevance also modulated responsiveness in affective or emotional regions of the brain [30].

Different activation profiles were associated with other core regions of the default network, such as the dorsal medial prefrontal cortex (dmPFC), the middle temporal gyri (MTG) and the hippocampal formation. The dmPFC and the bilateral lateral MTG were engaged only in high personal relevance scenarios relative to both medium and low personal relevance scenarios (high relevance>medium relevance∼low relevance) (Tables S3 and S4 in SI). Dorsal and ventral regions of the mPFC (Figure 3) have been proposed to subserve different top-down systems in self-relevance appraisal [31], [32]. The ventral system is postulated to mediate the “identification and appraisal of stimulus-induced self-relevance”, which fits with current findings in the ventral/amPFC regions [31]. In contrast, the dorsal system, which is known to be involved in inference processing [33], is held to mediate “cognitive control in the generation of explicitly self-referential decisions” [31]. This would imply that control processes, such as evaluation, introspection and recollection arising from associative brainstorming, are additionally and selectively involved when processing contexts involving friends or family.

**Figure 4:**
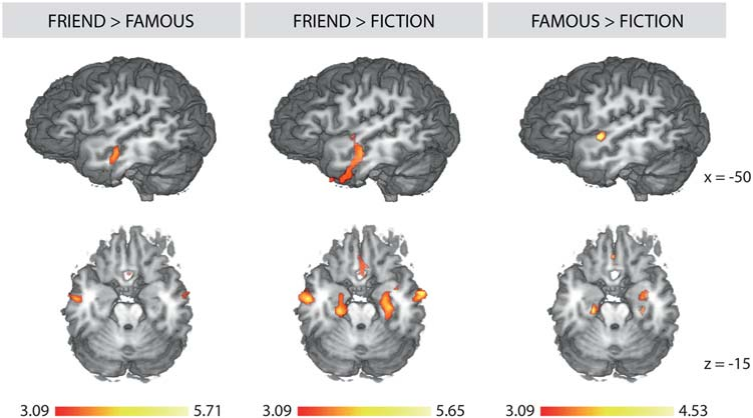
Other Relevance Patterns. Each column shows activations resulting from the indicated contrast. The left column shows results from the Friend>Famous contrast (inclusive mask: Friend>Control), the middle column shows results from the Friend>Fiction contrast (inclusive mask: Friend>Control) and the right column shows results from the Famous>Fiction contrast (inclusive mask: Famous>Control). All reported activations were corrected for multiple comparisons (p<.05) by employing cluster-size thresholding using Monte-Carlo simulations (initial height threshold: z = 3.09). The top row shows the activation profile in the left lateral temporal gyri whereas the bottom row depicts the activation profile in the bilateral hippocampal formation across contrasts.

The lateral MTG (Figure 4), on the other hand, is held to underlie representations of semantic details associated with recollected autobiographical knowledge [10]. That this region is most strongly engaged when processing friend scenarios is fitting given that we undoubtedly have a wider extent of autobiographical semantic knowledge concerning our friends and family compared to famous people or fictional characters.

Regions in the bilateral hippocampal formation (Figure 4), in contrast, were equivalently engaged during high and medium personal relevance contexts. Furthermore, these regions were more strongly activated during both high and medium personal relevance contexts compared to low personal relevance contexts (high relevance∼medium relevance>low relevance) (Figure 4, Table S2 and S3 in SI). The hippocampal formation is known to be involved in the encoding, retention and retrieval of episodic or event memories [34]–[36]. The recruitment of these regions in the present context may thus reflect the processing of information that is generally more salient in one's daily life as the high and medium relevance entities are likely to be associated with a greater extent of immediately accessible episodic memories.

It should be noted that the experimental design was such that correct answers for the four friend-real and famous-real conditions were the same across context types (Fig 1A: Possible? - Yes; Impossible? - No). The two fiction conditions differed in this respect as the fiction-interactive condition was the only experimental condition in which the correct responses were the other way round (Fig 1A: Possible? - No; Impossible? - Yes). The lower task conflict associated with the real conditions relative to the fiction conditions may therefore be another factor that contributed to the differences that surfaced when comparing the real conditions to the fiction conditions. This issue however does not affect the comparison between high relevance (friend) versus medium relevance (famous) real entities.

### Wider implications of findings

That core regions of the brain's default network are spontaneously modulated by the degree of stimulus-associated personal relevance is a consequential finding for two reasons. Firstly, the findings suggest that one of the factors that guide our implicit knowledge of what is real and unreal is the degree of coded personal relevance associated with a particular entity/character representation.

How is this operationalized? Our proposal is that when we encounter information concerning an entity/character, the conceptual knowledge that we possess in relation to this person is spontaneously activated. Our conceptual knowledge in relation to real people is far more extensive and multifaceted compared to that of fictional entities. For instance, the kind of associations most people have for a fictional character such as Cinderella (evil stepfamily, glass slipper, fairy godmother, the handsome prince, midnight, etc.) are limited to the context of the story in which we learnt about her. In comparison, our associations about a real famous entity such as George Bush is far more wide-ranging (his appearance, his position in the social hierarchy, my personal feelings towards him, my knowledge regarding the feelings of others towards him, his politics, his team, his family, the degree of influence he has on my life, the last time I saw him on TV, etc.). Our associations for people we know personally are even broader and richer than that for famous people.

The engagement of the amPFC and the PCC as a function of personal relevance reflects the retrieval, coordination and integration of such multidimensional and relationally complex information. These include autobiographical, episodic and self-referential information which are automatically accessed with the introduction of a familiar entity. The more relevant the person is to oneself, the wider the range of stored information associated with that person and, consequently, the greater the amount of information that is automatically retrieved and integrated when presented with appropriate stimuli [8].

What this might translate to at a phenomenological level is that a real person feels more “real” to us than a fictional character because we automatically have access to far more comprehensive and multi-flavored conceptual knowledge in relation to the real people than fictional characters. This would also explain why a real person we know personally (a friend) feels more real to us than a real person who we do not know personally (George Bush).

Indeed, there is evidence from developmental psychology that even children tend to evaluate the factual nature of fictional stories based on how the events therein fit with their own world knowledge. For instance, parents reported that their 4-year old children consider fictional characters that are associated with specific regular events in one's life, such as Santa Claus, the Easter Bunny and the Tooth Fairy, to be more real than fictional characters that are not related to real-life events, such as dragons, fairies and monsters [37]. It has also been shown that children use contextual information when making a decision about the reality status of a novel entity [38]. For instance, 5-year-olds judge novel entities to be real more often when they encounter them in real everyday or scientific contexts compared to fantastical contexts. Such findings indicate the modulatory role of factors such as our personal experience in understanding the distinction between reality and fiction.

With respect to the generalizability of the findings, it is important to note that personal relevance may not be unequivocally associated with what is real (relative to what is unreal). After all, fictional realms can also be associated with a high degree of personal relevance in certain contexts, such as in chronic computer gaming and religiosity. For instance, it may be possible that under certain circumstances a fictional entity of high personal relevance (e.g., a “World of Warcraft” character to a chronic gamer) could yield greater activation than a real famous person of low personal relevance (for instance, a famous talk show host to the same person). We believe that such situations in which the phenomenological aspect of the reality-fiction line is somewhat blurred provide rich ground for further investigation.

An additional point to keep in mind is that the concept of “personal relevance” may not be entirely synonymous with that of “self relevance”. The customary use of the word “self” indicates a direct link to one's self concept – knowledge of one's abilities and skills, one's personality attributes, etc. The object in question in such cases is the “Self” or the “I” (for instance, “Does this word describe you?” as opposed to “Does this word describe your mother?” or “Does this word describe Cinderella?”). We therefore suggest that adopting the term “personal relevance” would be necessary and fruitful for future work in this field because this term more accurately captures the wider connotation of what is meant by the phenomenon in question.

Secondly, given that we are predisposed to automatically attend to and further process self relevant information [39]–[41], the current results lend support to recent proposals that the default network is activated when engaging in mental simulation or “imaginative constructions of hypothetical events or scenarios” [17]. What is particularly noteworthy is that differences in the activation of this network in the present study were demonstrated even when comparing conditions that were not significantly different on any of the behavioral task measures (friend∼famous, Figure 2). This overrules the argument that the observed differential activity within the default network is simply attributable to low task load.

The overarching function of the default network is a matter of some debate [19], [42], [43]. While some findings indicate that the network is primarily recruited during task-irrelevant or “stimulus-independent thought”, there is evidence to suggest that this network is also responsive when monitoring the external environment for task relevant stimuli or during “stimulus-oriented thought”. The current findings argue against this dichotomy and suggest that a middle ground is likely to be at play as we show that stimulus-dependent information spontaneously triggered the default network beyond the pure task context. In other words, as the personal relevance associated with the stimulus was not task-relevant in the present study, the findings speak for the stimulus-dependent triggering of task-independent thought. This fits well with contemporary proposals that highlight the anticipatory nature of the brain and the default network's role in this capacity by which associations, analogies and predictions are automatically evoked about what is likely to be relevant in a given situation when faced with novel input [44], [45]. It is to be noted that it is not possible to fully exclude that the current findings are relevant primarily for the individual brain regions in question and not the network as a whole. However, co-engagement of default network regions outside the mPFC and PCC argue against this conclusion. As such then, the current findings constitute evidence for a novel approach (by varying the semantic context but not the demands of the task, as in the friend versus famous comparisons) that holds much promise for further study of the functional relevance of the default network.

## Materials and Methods

### Participants

The final sample included 19 right-handed healthy volunteers (10 female; mean age: 24.58; age range: 21–30) with normal or corrected-to-normal visual acuity. 2 participants were excluded from the original pool of subjects due to poor behavioral performance (less than 70% correct responses in one or more of the conditions). The participants were native German speakers with no reported history of neurological or psychiatric illness. None of the participants were taking medication at the time of measurement. All gave informed consent orally before participation.

### Ethics Statement

The experimental standards were approved by the local ethics committee of the University of Leipzig in Germany.

### Experimental design

A 3×2 repeated measures factorial design was employed with 40 trials per experimental condition. One factor was the character type (friend-real, famous-real, fictional) and the second factor reflected the context type (imaginative, interactive). The experimental conditions together with a control condition (40 trials) and 20 resting baseline trials were presented in a randomized trial design. With a trial length of 8 seconds and total of 300 trials, the experimental session lasted 40 minutes. The participants were given task instructions and performed a 5-minute practice session on a laptop prior to the fMRI session. After the experiment, the subjects were debriefed and requested to fill out a feedback form.

### Experimental Task

Verbal one-sentence scenarios were presented in which a real protagonist is involved in different contexts with a character that was either a fictional character (e.g., Cinderella), a real person who is world famous (e.g., George Bush) or a real person who was either a friend or family of the participant (e.g., the participant's mother).

Participants were asked two weeks prior to the experiment to submit a list of 11 names of their close friends and family. They were also asked to read through a list containing names of 11 fictional characters and a list containing names of 11 famous people, and to indicate whether they were familiar with all the characters and people in the list. 10 names per list type (friend/famous/fictional) were used as stimuli within the fMRI study. The remaining 1 name per list was used as stimuli during the practice trials.

The contexts were either Imaginative Contexts (dreamt about – *geträumt von*, thought about – *gedacht an*, remembered – *erinnert an*, pondered about – *nachgedacht über*) or Interactive Contexts (greeted - *gegrüßt*, dined with – *gegessen mit*, conversed with – *unterhalten mit*, discussed with - *diskutiert mit*). The participants' task was to evaluate whether each scenario was possible or not (Figure 1) by determining whether the event it portrayed could occur given the physical reality of our world.

Within this framework it would be, for instance, possible that someone thought about George Bush or Cinderella or the participant's mother. However, while it could be the case that someone interacted with a real person such as George Bush or the participant's mother, it would be factually impossible that s/he interacted with a fictional character such as Cinderella. To ensure that participants would have to make an equal number of “yes” and “no” responses when making a decision, a question cue (“Possible?” or “Impossible?”) was presented to the participants after each scenario sentence to which they had to prepare the appropriate “yes” or “no” response (Figure 1). For instance, if the question cue “Possible?” followed the scenario “Someone spoke to Cinderella yesterday”, the correct response would involve pressing the “no” button. If, instead, the question cue “Impossible?” followed the same scenario, the correct response would involve pressing the “yes” button.

In the control condition, participants made judgments concerning the two response keys (Figure 1). During the course of the experiment, the left button press was always used to indicate “yes” as an answer to the question cue and the right button press always signaled a “no” response. In line with these response codes, the control condition statements were devised to be either true (e.g., The “yes” button is the left button) or false (e.g., The “yes” button is the right button). The trial events of the control conditions were made comparable to the experimental conditions by having one of two question cues (“True?” or “False?”) follow such statements to which the participants were required to accurately respond. The behavioral measures obtained for all 7 conditions included reaction time (RT), percentage of correct responses (PCR) and perceived difficulty. The latter was obtained during the post-fMRI feedback session where subjects were asked to report how difficult they perceived each of the conditions to be on a scale of 1–7 (1: very easy, 7: very difficult).

During the feedback session, participants were asked to indicate whether any of the famous people and fictional characters had any special relevance for them (examples for such indications included having collections of any sort in relation to any character or special memories associated with any character). Only two participants reported a special association for a fictional character. In addition, just as in our previous study, participants were also asked to indicate if they had interacted in the past with any of the famous people in real life. Only one participant reported having interacted with one of the famous entities. The pattern of findings remained the same even when repeating the analyses after excluding all trials involving this entity for this participant.

### MRI scanning procedure

The imaging was carried out on a 3 T Bruker (Ettlingen, Germany) Medspec 30/100 system, which was equipped with the standard birdcage head coil. Participants were placed on the scanner bed in a supine position with their right index and middle fingers positioned on the appropriate response buttons of a 2-button response box. The participants' hands were carefully stabilized and form-fitting cushions were used to prevent head, arm and hand movements. Earplugs were also provided to the participants so that scanner noise would be attenuated. The sentences were presented using the VisuaStim Digital MRI Video System (Resonance Technology, Northridge, USA), which is a high-resolution visor (800×600 resolution) comprising of two small TFT-screens placed close to the eyes.

24 axial slices (19.2 cm field of view; 64×64 pixel matrix; 4 mm thickness; 1 mm spacing; in-plane resolution of 3×3 mm) parallel to bicommissural line (AC-PC) covering the whole brain were acquired using a single-shot gradient echo-planar imaging (EPI) sequence (TR = 2000 ms; TE = 30 ms; flip angle = 90°; acquisition bandwidth = 100 kHz) sensitive to blood oxygenation level-dependent contrast. Prior to the functional imaging, 24 anatomical T1-weighted MDEFT images (data matrix = 256×256; TR = 1300 ms; TI = 650 ms TE = 10 ms) with the same spatial orientation as the functional data were acquired.

### fMRI data analysis

The fMRI data were processed using the LIPSIA software package [46], which contains tools for preprocessing, registration, statistical evaluation and presentation of fMRI data. Functional data were first motion-corrected using a matching metric based on linear correlation. To correct for the temporal offset between the slices acquired in one scan, a sinc-interpolation based on the Nyquist-Shannon-Theorem was applied. Low-frequency signal changes and baseline drifts were removed using a temporal highpass filter with a cut-off frequency of 1/120 Hz. Spatial smoothing was performed with a Gaussian filter of 5.65 mm FWHM.

To align the functional data slices onto a three-dimensional stereotactic coordinate reference system, a rigid linear registration was performed with 6 degrees of freedom (3 rotational, 3 translational). The rotational and translational parameters were acquired on the basis of the MDEFT [47], [48] slices to achieve an optimal match between these slices and the individual three-dimensional reference data set. This high-resolution three-dimensional reference data set was acquired for each subject during a previous scanning session. The MDEFT volume data set with 160 slices and 1 mm slice thickness was standardized to the Talairach stereotactic space [49]. These rotational and translational parameters were subsequently normalized in that they were transformed by linear scaling to a Talairach standard size. The normalized parameters were then used to transform the functional slices using trilinear interpolation so that the resulting functional slices were aligned with the stereotactic coordinate system, thus generating output data with a spatial resolution of 3×3×3 mm (27 cubic mm).

The statistical evaluation was based on a least-squares estimation using the general linear model for serially autocorrelated observations [50], [51]. The design matrix used for modelling the data consisted of onset vectors for the correct trials of each of the seven conditions (6 experimental, 1 control), with additional vectors for empty trials and cued response periods which included trial-by-trial RT as a parameter. The design matrix was generated with a box-car function, convolved with the hemodynamic response function. Brain activations were analyzed in an event-related design, time-locked to the presentation of the scenario of all presented trials. The model equation, including the observation data, the design matrix, and the error term, was convolved with a Gaussian kernel dispersion of 4 sec FWHM to account for the temporal autocorrelation [51]. In the following, contrast images or beta value estimates of the raw-score differences between specified conditions were generated for each participant. As all individual functional data sets were aligned to the same stereotactic reference space, the single-subject contrast images were entered into a second-level random-effects analysis for each of the contrasts. One-sample t tests were employed for the group analyses across the contrast images of all subjects which indicated whether observed differences between conditions were significantly distinct from zero. *t* values were subsequently transformed into *z* scores.

Cluster-wise control of family-wise error corrections were carried out to deal with the multiple comparison issue. Corrections (*P*<0.05) were carried out using a combination of individual voxel probability thresholding (*Z* = 3.09) and minimum cluster-size thresholding (999 cubic mm) as computed using Monte-Carlo simulations [52], [53].

Inclusive mask analyses were carried out from the corrected one-sample *t*-tests. In each inclusive mask analysis, the statistic parametric map of the random-effects analysis of the experimental condition A-versus-control condition direct contrast was used as an inclusive mask in the random-effects analysis of the experimental condition A-versus-experimental condition B direct contrast. The findings that result from an inclusive masked analysis indicate which brain areas were significantly activated for experimental condition A relative to experimental condition B, but only if the same regions were also more highly activated in experimental condition A relative to a control condition C.

## Supporting Information

Supporting Results S1 (0.05 MB DOC)

Table S1 Descriptive data (mean and standard deviation) of the behavioral measures (RT: Reaction Time, PCR: Percentage of correct responses, Perceived Difficulty) for all conditions: friend-imaginative, friend-interactive, famous-imaginative, famous-interactive, fiction-imaginative, fiction-interactive, and control
(0.04 MB DOC)

Table S2 List of activations from the Famous>Fiction inclusive mask contrast (Mask: Famous>Control). Cluster-wise control of family-wise error (p<0.05) was carried out to correct for multiple comparisons.
(0.04 MB DOC)

Table S3 List of activations from the Friend>Fiction inclusive mask contrast (Mask: Friend>Control). Cluster-wise control of family-wise error (p<0.05) was carried out to correct for multiple comparisons.
(0.06 MB DOC)

Table S4 List of activations from the Friend>Famous inclusive mask contrast (Mask: Friend>Control). Cluster-wise control of family-wise error (p<0.05) was carried out to correct for multiple comparisons.
(0.04 MB DOC)
