# Supplementary material for: Reality = Relevance? Insights from Spontaneous Modulations of the Brain's Default Network when Telling Apart Reality from Fiction
Source: PLoS One. 2009 Mar 11;4(3):e4741. doi: 10.1371/journal.pone.0004741 (PMC2648967; doi:10.1371/journal.pone.0004741)
Supplement: Supporting Results S1 — (0.05 MB DOC) [file pone.0004741.s001.doc]

**SUPPORTING RESULTS**

***Behavioral findings: RT***

A 3 x 2 repeated measures ANOVA was carried out on the response variable RT with Factor 1: Entity Type having three levels (friend, famous, fiction) and Factor 2: Context Type having two levels (imaginative, interactive). There were significant main effects of entity type (*F*2, 17 = 109.44, *P* < .001; partial eta squared/hp2 = 0.899) and context type (*F*1, 18 = 12.65, *P* < .005, hp2 = 0.413) as well as a significant interaction effect between the two factors (*F*2, 17 = 7.52, *P* < .005, hp2 = 0.295). The main effects indicate that the participants were slower when responding to fictional scenarios compared to famous scenarios (*F*1, 18 = 107.75, *P* < .001, hp2 = 0.857) and friend scenarios (*F*1, 18 = 160.53, *P* < .001, hp2 = 0.899), but there was no significant difference between the RT for famous versus friend scenarios (*F*1, 18 = 3.82, *P* > .05). Further analyses of the interaction effect indicated that participants took significantly longer to respond to interactive than imaginative contexts in the case of fiction scenarios relative to both friend (*F*1, 18 = 9.77, *P* < .01, hp2 = 0.352) and famous contexts (*F*1, 18 = 8.32, *P* < .05, hp2 = 0.316). There were no significant differences between the RT for different contexts when comparing the famous and friend scenarios (*F*1, 18 = 0.28, *P* > .05).

Paired samples two-tailed t-tests were carried out between the experimental conditions and the control condition. Compared to the RT for the control condition statements, participants responded faster to friend-imaginative scenarios (*t*_18_ = -5.47, *P* < .001), friend-interactive scenarios (*t*_18_ = -5.27, *P* < .001), famous-imaginative scenarios (*t*_18_ = -4.42, *P* < .001), and famous-interactive scenarios (*t*_18_ = -4.19, *P* < .005). The differences between the control condition and the fiction-imaginative scenarios (*t*_18_ = -2.12, *P* > .05) and fiction-interactive scenarios (*t*_18_ = 1.5, *P* > .05) were non-significant.

***Behavioral findings: PCR***

The 3 x 2 repeated measures ANOVA on response accuracy revealed a significant main effect of entity type (*F*2, 17 = 23.80, *P* < .001; partial eta squared/hp2 = 0.569), but not context type (*F*1, 18 = 3.24, *P* >.05), as well as a significant interaction effect between the two factors (*F*2, 17 = 3.83, *P* < .05, hp2 = 0.175). The main effects of the repeated measures ANOVA showed that the response accuracy was lower when responding to fictional scenarios compared to famous scenarios (*F*1, 18 = 22.03, *P* < .001, hp2 = 0.55) and friend scenarios (*F*1, 18 = 29.16, *P* < .001, hp2 = 0.618), but there was no significant difference between the PCRs of famous and friend scenarios (*F*1, 18 = 2.4, *P* > .05). Further analyses of the interaction effect indicated that participants were significantly less accurate when responding to interactive than imaginative contexts in the case of fiction scenarios relative to friend scenarios (*F*1, 18 = 4.88, *P* < .04, hp2 = 0.213). There were no significant differences between the response accuracy for different contexts when comparing the famous and friend scenarios (*F*1, 18 = 0.76, *P* > .05) or the fiction and famous scenarios (*F*1, 18 = 4.21, *P* > .05).

Paired samples t-tests revealed that compared to the response accuracy for the control condition statements, participants responded more accurately to friend-imaginative scenarios (*t*_18_ = 3.83, *P* < .005), friend-interactive scenarios (*t*_18_ = 4.11, *P* < .005), famous-imaginative scenarios (*t*_18_ = 3.20, *P* < .01), and famous-interactive scenarios (*t*_18_ = 2.93, *P* < .01). The differences between the control condition and the fiction-imaginative scenarios (*t*_18_ = 0.77, *P* > .05) fiction-interactive scenarios (*t*_18_ = -0.96, *P* > .05) were non-significant.

***Behavioral findings: Perceived Difficulty***

Non-parametric tests (Wilcoxon Signed Ranks Test) were carried out between the conditions on the Perceived Difficulty response measure as this was a non-continuous variable. All the results (2-tailed, exact significance) are given below in tabular form.

|  | *FRIEND interactive* | *FAMOUS*  *imaginative* | *FAMOUS*  *interactive* | *FICTION*  *imaginative* | *FICTION*  *interactive* | *CONTROL* |
| --- | --- | --- | --- | --- | --- | --- |
| *FRIEND*  *imaginative* | P > .05  Z = 0.65 | P > .05  Z = 1.52 | P > .05  Z = 1.73 | **P < .001**  **Z = 3.25** | **P < .001**  **Z = 3.52** | **P < .001**  **Z = 3.48** |
| *FRIEND interactive* | -- | P > .05  Z = 0.17 | P > .05  Z = 1.89 | **P < .05**  **Z = 2.45** | **P < .001**  **Z = 3.48** | **P < .001**  **Z = 3.47** |
| *FAMOUS*  *imaginative* |  | -- | P > .05  Z = 1.39 | **P < .001**  **Z = 3.25** | **P < .001**  **Z = 3.38** | **P < .001**  **Z = 3.46** |
| *FAMOUS*  *interactive* |  |  | -- | **P < .05**  **Z = 2.01** | **P < .001**  **Z = 3.48** | **P < .001**  **Z = 3.46** |
| *FICTION*  *imaginative* |  |  |  | -- | P > .05  Z = 0.19 | **P < .01**  **Z = 2.62** |
| *FICTION*  *interactive* |  |  |  |  | -- | P > .05  Z = 1.83 |

***fMRI findings***

No brain region was found to significantly activated as a function of context type (Direct contrasts: Interactive > Imaginative; Imaginative > Interactive). Contrasts of context within entity type revealed that no regions were significantly activated when comparing the famous-real interactive and imaginative conditions (Direct contrasts: Famous Interactive > Famous Imaginative; Famous Imaginative > Famous Interactive), nor the friend-real interactive and imaginative conditions (Direct contrasts: Friend Interactive > Friend Imaginative; Friend Imaginative > Friend Interactive). While no brain region was found to be activated for the Fiction Imaginative condition relative to the Fiction Interactive condition (Direct contrast: Friend Imaginative > Friend Interactive), three regions of the brain were found to be more active for the Friend Interactive condition relative to the Friend imaginative condition (Direct contrast: Friend Interactive > Friend Imaginative). These regions included the dorsal inferior parietal cortex (28, -71, 42; 2835 cubic mm), the left dorsal premotor cortex (-26, -2, 54; 1377 cubic mm), the right dorsal premotor cortex and adjoining posterior superior frontal gyrus (19, 7, 42; 1080 cubic mm).

In addition, no brain areas were found to be significantly activated when carrying out inclusive mask analyses of the fiction conditions with friend-real conditions (Mask: Fiction > Control), and the fiction conditions with the famous-real conditions (Mask: Fiction > Control). The previously reported regions associated with fiction relative to the famous scenarios (Abraham et al., 2007), namely aspects of the left lateral inferior frontal gyrus (BA 45, 47), were only found in the direct contrasts of the fiction and the real conditions (Fiction > Famous & Fiction > Friend).
