# Supplementary material for: Reality = Relevance? Insights from Spontaneous Modulations of the Brain's Default Network when Telling Apart Reality from Fiction
Source: PLoS One. 2009 Mar 11;4(3):e4741. doi: 10.1371/journal.pone.0004741 (PMC2648967; doi:10.1371/journal.pone.0004741)
Supplement: Table S1 — Descriptive data (mean and standard deviation) of the behavioral measures (RT: Reaction Time, PCR: Percentage of correct responses, Perceived Difficulty) for all conditions: friend-imaginative, friend-interactive, famous-imaginative, famous-interactive, fiction-imaginative, fiction-interactive, and control (0.04 MB DOC) [file pone.0004741.s002.doc]

| **TABLE S1** | RT (ms) | | PCR (%) | | Perceived Difficulty | |
| --- | --- | --- | --- | --- | --- | --- |
| Conditions | Mean | SD | Mean | SD | Mean | SD |
| Friend- Imaginative | 789.21 | 143.49 | 97.46 | 3.09 | 1.32 | 0.58 |
| Friend- Interactive | 800.16 | 138.42 | 97.76 | 2.87 | 1.53 | 1.02 |
| Famous- Imaginative | 801.79 | 132.57 | 96.71 | 3.73 | 1.58 | 0.90 |
| Famous-Interactive | 806.21 | 144.30 | 96.71 | 3.34 | 1.79 | 1.18 |
| Fiction-Imaginative | 850.79 | 164.28 | 91.69 | 7.23 | 2.68 | 1.16 |
| Fiction-  Interactive | 915.68 | 131.69 | 88.07 | 10.21 | 3.26 | 1.56 |
| Control | 890.32 | 144.95 | 90.31 | 2.20 | 4.11 | 2.28 |
