# Supplementary material for: Reality = Relevance? Insights from Spontaneous Modulations of the Brain's Default Network when Telling Apart Reality from Fiction
Source: PLoS One. 2009 Mar 11;4(3):e4741. doi: 10.1371/journal.pone.0004741 (PMC2648967; doi:10.1371/journal.pone.0004741)
Supplement: Table S2 — List of activations from the Famous>Fiction inclusive mask contrast (Mask: Famous>Control). Cluster-wise control of family-wise error (p<0.05) was carried out to correct for multiple comparisons. (0.04 MB DOC) [file pone.0004741.s003.doc]

| **TABLE S2** | x | y | z | BA | mm^3^ | Z-max |
| --- | --- | --- | --- | --- | --- | --- |
| Anterior medial PFC / ACC | -5 | 49 | 0 | 10/32 | 7587 | 4.19 |
| Ventral medial PFC / ACC | -5 | 40 | -9 | 11/32/10 | .. | 3.98 |
| ACC | -2 | 28 | -12 | 32/25 | .. | 4.54 |
| Subgenual ACC | -2 | 13 | -6 | 25 | .. | 4.38 |
| PCC/RSC | -8 | -59 | 21 | 31/30/29 | 2457 | 4.49 |
| Superior temporal gyrus | 52 | -5 | 0 | 22 | 2160 | 4.52 |
| Insula | 37 | -5 | -3 | - | .. | 4.01 |
| Superior temporal gyrus | -53 | -5 | 0 | 22 | 1485 | 4.33 |
| HF/PHG | 25 | -26 | -12 | - | 1647 | 4.13 |
| HF/Amygdala | 28 | -14 | -15 | - | .. | 3.88 |
| HF/PHG | -23 | -26 | -15 | - | 1161 | 4.20 |
| PHG | -14 | -23 | -21 | 19 | .. | 3.71 |

Abbreviations: ACC-anterior cingulate cortex, HF-hippocampal formation, PCC-posterior cingulate cortex, PFC–prefrontal cortex, PHG-parahippocampal gyrus, RSC–retrosplenial cortex
