# Supplementary material for: Reality = Relevance? Insights from Spontaneous Modulations of the Brain's Default Network when Telling Apart Reality from Fiction
Source: PLoS One. 2009 Mar 11;4(3):e4741. doi: 10.1371/journal.pone.0004741 (PMC2648967; doi:10.1371/journal.pone.0004741)
Supplement: Table S3 — List of activations from the Friend>Fiction inclusive mask contrast (Mask: Friend>Control). Cluster-wise control of family-wise error (p<0.05) was carried out to correct for multiple comparisons. (0.06 MB DOC) [file pone.0004741.s004.doc]

| **TABLE S3** | x | Y | z | BA | mm^3^ | Z-max |
| --- | --- | --- | --- | --- | --- | --- |
| Anterior medial PFC | -5 | 49 | 0 | 10/32 | 31725 | 5.65 |
| Ventral mPFC / ACC | -5 | 40 | 0 | 32/11/10 | .. | 5.59 |
| Anterior medial PFC/ACC | 7 | 46 | 0 | 10/32/11 | .. | 5.41 |
| Dorsal medial PFC | -2 | 43 | 24 | 9 | .. | 5.36 |
| Superior Frontal Gyrus | -14 | 37 | 36 | 8 | .. | 4.31 |
| Superior Frontal Gyrus | -17 | 49 | 27 | 9 | .. | 4.19 |
| Subgenual ACC | 1 | 13 | -3 | 25 | .. | 5.56 |
| Subgenual ACC | -5 | -2 | -9 | 25 | .. | 4.55 |
| PCC | -2 | -56 | 27 | 31/30/23/29 | 13095 | 5.66 |
| RSC | 13 | -53 | 9 | 29/30 | .. | 3.92 |
| RSC | -11 | -53 | 6 | 29/30 | .. | 5.33 |
| Middle temporal gyrus | 58 | -8 | -15 | 21 | 2700 | 5.01 |
| Inferior/Middle temporal gyrus | 49 | -8 | -27 | 20/21 | .. | 4.20 |
| Superior temporal gyrus | -50 | -11 | 3 | 22 | .. | 3.65 |
| Middle temporal gyrus | -53 | -17 | -9 | 21 | 6723 | 4.70 |
| Inferior/Middle temporal gyrus | -47 | -5 | -27 | 20/21 | .. | 4.10 |
| Temporal pole/MTG | -38 | 1 | -33 | 38/20 | .. | 4.11 |
| Temporal pole | -38 | 10 | -36 | 38 | .. | 3.75 |
| Temporal pole/MTG | 43 | 7 | -36 | 38/20 | 1107 | 3.93 |
| HF/Amygdala | 25 | -23 | -9 | - | 5049 | 5.07 |
| HF/PHG | 19 | -20 | -21 | - | .. | 4.44 |
| Inferior temporal gyrus | 34 | -2 | -21 | 20 | .. | 4.24 |
| HF/PHG | -17 | -26 | -21 | - | 4725 | 4.74 |
| PHG | -20 | -44 | -6 | 19 | .. | 4.53 |

Abbreviations: ACC-anterior cingulate cortex, HF-hippocampal formation, MTG-Middle Temporal gyrus, PCC-posterior cingulate cortex, PFC–prefrontal cortex, PHG-parahippocampal gyrus, RSC–retrosplenial cortex
