# Supplementary material for: Reality = Relevance? Insights from Spontaneous Modulations of the Brain's Default Network when Telling Apart Reality from Fiction
Source: PLoS One. 2009 Mar 11;4(3):e4741. doi: 10.1371/journal.pone.0004741 (PMC2648967; doi:10.1371/journal.pone.0004741)
Supplement: Table S4 — List of activations from the Friend>Famous inclusive mask contrast (Mask: Friend>Control). Cluster-wise control of family-wise error (p<0.05) was carried out to correct for multiple comparisons. (0.04 MB DOC) [file pone.0004741.s005.doc]

| **TABLE S4** | x | y | z | BA | mm^3^ | Z-max |
| --- | --- | --- | --- | --- | --- | --- |
| Anterior medial PFC | -5 | 40 | 3 | 10/32 | 18549 | 5.13 |
| Ventral medial PFC | -5 | 49 | 0 | 11/10/32 | .. | 5.13 |
| ACC | 7 | 37 | 0 | 32 | .. | 4.65 |
| ACC | 1 | 31 | 9 | 32 | .. | 4.84 |
| Dorsal medial PFC | -8 | 40 | 45 | 9/8 | .. | 3.66 |
| Subgenual ACC | 1 | 10 | -9 | 25 | 2322 | 4.53 |
| PCC | -5 | -56 | 30 | 31/30/23 | 13014 | 5.72 |
| RSC | -11 | -53 | 3 | 29/30 | .. | 5.19 |
| Middle temporal gyrus | 49 | -11 | -24 | 21 | 1134 | 4.11 |
| Middle temporal gyrus | -53 | -14 | -15 | 21 | 1404 | 4.34 |

Abbreviations: ACC-anterior cingulate cortex, HF-hippocampal formation, PCC-posterior cingulate cortex, PFC–prefrontal cortex, PHG-parahippocampal gyrus, RSC–retrosplenial cortex
